# Current irritability associated with hastened depressive recurrence and delayed depressive recovery in bipolar disorder

**DOI:** 10.1186/s40345-016-0056-2

**Published:** 2016-07-30

**Authors:** Laura D. Yuen, Saloni Shah, Dennis Do, Shefali Miller, Po W. Wang, Farnaz Hooshmand, Terence A. Ketter

**Affiliations:** Department of Psychiatry and Behavioral Sciences, Stanford University School of Medicine, 401 Quarry Road, Stanford, CA 94305-5723 USA

**Keywords:** Irritability, Bipolar disorder, Bipolar depression, Recurrence, Recovery, Longitudinal

## Abstract

**Background:**

Current irritability is associated with greater retrospective and current bipolar disorder (BD) illness severity; less is known about prospective longitudinal implications of current irritability. We examined relationships between current irritability and depressive recurrence and recovery in BD.

**Methods:**

Outpatients referred to the Stanford BD Clinic during 2000–2011 were assessed with the Systematic Treatment Enhancement Program for BD (STEP-BD) Affective Disorders Evaluation at baseline, and with the Clinical Monitoring Form during follow-up during up to 2 years of naturalistic treatment. Prevalence and clinical correlates of any current irritability in depressed and recovered (euthymic ≥8 weeks) BD patients were assessed. Kaplan–Meier analyses (Log-Rank tests) assessed relationships between current irritability and longitudinal depressive severity, with Cox Proportional Hazard analyses assessing potential mediators.

**Results:**

Recovered BD outpatients with vs. without current irritability had significantly higher rates of 13/19 (68.4 %) other baseline unfavorable illness characteristics/current mood symptoms and hastened depressive recurrence (Log-Rank *p* = 0.020), driven by lifetime history of anxiety disorder and prior year rapid cycling, and attenuated by history of psychosis. Depressed BD outpatients with vs. without current irritability had significantly higher rates of 7/19 (36.8 %) other unfavorable illness characteristics/current mood symptoms and delayed depressive recovery (Log-Rank *p* = 0.034), NOT mediated by any assessed parameter.

**Limitations:**

Limited generalizability beyond our predominately white, female, educated, insured American BD specialty clinic sample.

**Conclusions:**

Current irritability was associated with hastened depressive recurrence and delayed depressive recovery in BD. Treatment studies targeting irritability may yield strategies to mitigate increased longitudinal depressive burden.

## Background

Bipolar disorder (BD) is a chronic illness associated with high rates of recurrence and impaired functionality (Ketter 2010; Solomon et al. 1995). Up to one-half of BD patients have mood episode recurrence within 1 year of recovery (Solomon et al. 1995), commonly with severe consequences, including higher rates of non-response, social morbidity, and impaired functioning (Berk et al. 2011; Lish et al. 1994; Rosa et al. 2012). Although mood elevation episodes define BD, bipolar depression is more pervasive (Judd et al. 2002, 2003), and has been associated with functional impairment (Goldberg and Harrow 2011; Gyulai et al. 2008) and suicidality (Dilsaver et al. 1997; Suttajit et al. 2013), with the latter present in almost four fifths of depressed patients (Dilsaver et al. 1997). Notably, even subsyndromal depressive symptoms can undermine function (Altshuler et al. 2006; Goldberg and Harrow 2011). In addition, bipolar depression negatively impacts BD illness course; with illness severity worsening with recurrent episodes, independent of treatment (Maj et al. 1992).

Previous studies have demonstrated current irritability to be both highly prevalent and indicative of worse illness severity in acute bipolar depression. Irritability is a core symptom of depression in children and adolescents (but not in adults) (American Psychiatric Association 2013), and is present in up to three quarters of depressed BD patients (Winokur et al. 1969). Current irritability has been associated with multiple unfavorable illness characteristics in bipolar depression, including earlier onset age and higher rates of suicidal ideation, axis I comorbidity, atypical depressive features, and depressive mixed states (Balázs et al. 2006; Benazzi and Akiskal 2005; Benazzi et al. 2004). Additionally, current irritability appears closely related to current anxiety and lifetime history of anxiety disorder in BD (Yuen et al. 2016).

The negative longitudinal impact of current mood symptoms is well established (Maj et al. 2003; Perlis et al. 2006). Residual affective symptoms (which may be related to irritability) in recovered patients predicted hastened episode recurrence (Perlis et al. 2006). Also, among depressed bipolar I disorder patients, those with agitation (over half of whom had irritability) compared to those without agitation (only 15 % of whom had irritability) had significantly delayed depressive recovery (Maj et al. 2003). However, the association between current irritability and bipolar course requires further examination. In this prospective study, we examined longitudinal relationships between current irritability and depressive recurrence and recovery in bipolar disorder patients.

## Methods

Outpatients referred to the Stanford Bipolar Disorder Clinic from 2000 to 2011 were assessed using the Systematic Treatment Enhancement Program for BD (STEP-BD) Affective Disorders Evaluation (Sachs et al. 2002, 2003), which included the structured clinical interview for the fourth edition of the diagnostic and statistical manual of mental disorders (SCID for DSM-IV) (First et al. 1997) mood disorders module and the Clinical Global Impression for Bipolar Disorder-Overall Severity (CGI-BP-OS) score (Spearing et al. 1997). The Mini International Neuropsychiatric Interview (MINI) (Sheehan et al. 1998) was also performed to confirm bipolar and comorbid psychiatric disorder diagnoses. Patients were followed prospectively for up to 2 years, while receiving naturalistic treatment with a monthly modal visit frequency. The Clinical Monitoring Form (CMF) (Sachs et al. 2002) was used to assess clinical status at each follow-up visit. The Stanford University Administrative Panel on Human Subjects approved the STEP-BD protocol, as well as the subsequent similar Assessment, Monitoring, and Centralized Database protocol specific to Stanford University. Both protocols conformed to the standards of the Helsinki Declaration of 1975, and all subjects provided verbal and written informed consent before participating in the study.

Current irritability was assessed based on patient recall of the number of days among the 10 days prior to enrollment during which they experienced any irritability on the STEP-BD Affective Disorders Evaluation. For the primary analysis, we examined current irritability dichotomized according to the presence or absence of any irritability. For secondary analyses, we examined current irritability dichotomized according to the presence or absence of irritability for at least four and at least seven of the prior 10 days.

Trained medical and research staff collected data on 6 demographic parameters and 19 illness characteristics/current mood symptoms. The demographic parameters assessed were: (A) age (in years), (B) gender, (C) race/ethnicity, (D) education, (E) marital status, and (F) employment status. The illness characteristics/current mood symptoms assessed were: (1) lifetime anxiety disorder; (2) lifetime alcohol/substance use disorder; (3) lifetime eating disorder; (4) lifetime personality disorder; (5) bipolar II disorder; (5A) lifetime psychosis (which is very commonly associated with Bipolar I disorder); (5B) lifetime prior psychiatric hospitalization (which is also very commonly associated with bipolar I disorder); (6) ≥one first degree relative with mood disorder; (7) onset age (in years); (8) childhood (age <13 years) onset; (9) illness duration (in years); (10) Long illness duration (≥15 years); (11) episode accumulation (≥10 prior mood episodes); (12) lifetime suicide attempt; (13) rapid cycling in prior year; and (14) CGI-BP-OS; as well as current (i.e., any in the prior 10 days) (15) sadness; (16) anhedonia; (17) euphoria; (18) irritability; and (19) anxiety.

Statistical analyses were performed using Statistical Package for the Social Sciences (SPSS) Version 23.0 software (IBM Corp.; Armonk, NY, USA) on an Apple MacBook Air computer (Apple Corporation, Cupertino, CA, USA). Prevalence and clinical correlates of current irritability were examined in currently recovered (i.e., euthymic ≥8 weeks) and currently depressed (i.e., with a current major depressive episode) patients. Analytical statistics included Fisher’s Exact test comparisons of categorical data and independent-sample *t* test comparisons of continuous variables. In addition, binary logistic regression was used to adjust for potential confounding variables. Primary longitudinal analyses consisted of Kaplan–Meier survival analyses (Log-Rank tests), which compared times to depressive recurrence and recovery in patients with and without current irritability. We used the standard approaches of censoring patients with mood elevation prior to depressive recurrence in assessing time to depressive recurrence, and censoring patients with depressive prior to mood elevation recurrence in assessing time to mood elevation recurrence (Perlis et al. 2006). Secondary analyses included Cox proportional hazard analyses [hazard ratios (HRs) and 95 % confidence intervals (CIs)] of depressive recurrence and recovery, as well as of potential mediators of statistically significant longitudinal depressive illness severity findings. To select parameters for entry into mediator models, univariate Cox proportional hazard analyses were performed for all statistically significant clinical correlates of current irritability. Parameters with *p* < 0.05 were entered into a forward stepwise procedure, and covariates were included in the model if *p* < 0.05. Additionally, Cox proportional hazard analyses with time-dependent covariates were used to further characterize associations between current irritability and depressive recurrence and recovery. We used a two-tailed significance threshold with *p* < 0.05, with no correction for multiple comparisons.

## Results

Table 1 includes demographics, illness characteristics, and current mood symptoms of currently recovered and currently depressed patients, stratified by the presence or absence of any current irritability. In our overall sample of 503 outpatients with bipolar I disorder or bipolar II disorder, 105 (20.9 %) were currently recovered and 153 (30.4 %) were currently depressed. The prevalence of current irritability was nearly twice as high among currently depressed (68.6 %) vs. recovered (36.2 %, *χ*^2^ = 31.4, *df* = 1, *p* < 0.0001) patients.

**Table 1:** Demographics, illness characteristics, and current mood symptoms in recovered and depressed bipolar disorder outpatients with and without current irritability

	Currently recovered		Currently depressed	
	Current irritability	No current irritability	Current irritability	No current irritability
*N* (%)	38 (36.2)****	67 (63.8)	105 (68.6)****	48 (31.4)
Demographics				
A. Age (years, mean ± SD)	36.3 ± 14.0	35.9 ± 13.6	35.5 ± 13.2	37.8 ± 14.0
B. Female (%)	60.5	52.2	65.7	52.1
C. Caucasian (%)	69.4	80.3	87.6	85.4
D. College degree (%)	64.9	61.5	38.1**	66.7
E. Married (current, %)	42.1	33.3	38.1	35.4
F. Full-time employment (current, %)	40.5	30.3	29.5	17.0
Comorbid disorders (lifetime, %)				
1. Anxiety	60.5*	38.8	84.8**	60.4
2. Alcohol/Substance Use	71.1**	38.8	59.0	56.3
3. Eating	10.5	9.0	21.9	12.5
4. Personality	10.5	7.5	18.1	6.3
Other illness characteristics				
5. Bipolar II disorder (%)	57.9**	28.4	69.5**	43.8
* 5A. Psychosis (lifetime, %)*	*31.6*	55.2*	*32.4*	*43.8*
*5B. Psychiatric hospitalization (lifetime, %)*	*31.6*	55.2*	*21.0*	37.5*
6. ≥One 1° degree relative w mood disorder (%)	63.2*	37.3	65.7*	45.8
7. Onset age (years, mean ± SD)	17.0 ± 8.4*	20.8 ± 8.9	15.6 ± 6.3***	20.5 ± 9.5
8. Childhood (age <13 years) onset (%)	21.6	9.0	26.7	14.6
9. Illness duration (years, mean ± SD)	20.2 ± 16.1	15.8 ± 12.2	19.5 ± 13.8	17.0 ± 11.4
10. Long illness duration (≥15 years, %)	50.0	46.8	52.9	54.2
11. Episode accumulation (≥10, lifetime, %)	66.7*	42.4	78.1*	58.7
12. Suicide attempt (lifetime, %)	44.4**	16.7	32.7	37.5
13. Rapid cycling (prior year, %)	18.9*	4.8	31.1	25.0
14. CGI-BP-OS (current, mean ± SD)	2.6 ± 0.6***	1.9 ± 0.8	5.4 ± 0.8	5.3 ± 0.6
Current mood symptoms (any in prior 10 days, %)				
15. Sadness	31.6*	11.9	89.5	89.6
16. Anhedonia	23.7*	9.0	95.2	93.8
17. Euphoria	34.2****	1.5	34.3*	14.6
18. Irritability	100.0****	0.0	100.0****	0.0
19. Anxiety	57.9***	23.9	86.7*	68.8

*CGI-BP-OS* clinical global impression for bipolar disorder-overall severity, *SD* standard deviation

* *p* < 0.05, ** *p* < 0.01, *** *p* < 0.001, **** *p* < 0.0001 with vs.  Underline font indicates parameters with statistically significant relationships with current irritability. Italic font indicates parameters associated with bipolar I subtype. Missing data among recovered patients: 12.4 % for ≥10 prior episodes, 0.0–6.7 % for other parameters. Missing data among depressed patients: 7.2 % for ≥10 prior episodes, 0.0–1.3 % for other parameters

### Demographics and illness characteristics/current mood symptoms in recovered patients with vs. without current irritability

Among recovered patients, the presence compared to the absence of current irritability was significantly less common (36.2 vs. 63.8 % had and lacked current irritability, respectively, binomial test *p* < 0.0001). Nevertheless, recovered patients with vs. without current irritability had significantly higher rates lifetime anxiety (60.5 vs. 38.8 %, *χ*^2^ = 4.6, *df* = 1, *p* = 0.042) and alcohol/substance use (71.1 vs. 38.8 %, *χ*^2^ = 10.1, *df* = 1, *p* = 0.002) disorder, bipolar II disorder (57.9 vs. 28.4 %, *χ*^2^ = 8.9, *df* = 1, *p* = 0.004), having ≥one first degree relative with a mood disorder (63.2 vs. 37.3 %, *χ*^2^ = 6.5, *df* = 1, *p* = 0.015), episode accumulation (≥10 prior episodes, 66.7 vs. 42.4 %, *χ*^2^ = 5.0, *df* = 1, *p* = 0.031), lifetime suicide attempt (44.4 vs. 16.7 %, *χ*^2^ = 9.2, *df* = 1, *p* = 0.004), prior year rapid cycling (18.9 vs. 4.8 %, *χ*^2^ = 5.1, *df* = 1, *p* = 0.037), current sadness (31.6 vs. 11.9 %, *χ*^2^ = 6.1, *df* = 1, *p* = 0.020), anhedonia (23.7 vs. 9.0 %, *χ*^2^ = 4.3, *df* = 1, *p* = 0.047), euphoria (34.2 vs. 1.5 %, *χ*^2^ = 22.5, *df* = 1, *p* < 0.0001), irritability (by definition 100.0 vs. 0.0 %, *χ*^2^ = 105.0, *df* = 1, *p* < 0.0001), and anxiety (57.9 vs. 23.9 %, *χ*^2^ = 12.2, *df* = 1, *p* = 0.0007), as well as earlier onset age (17.0 ± 8.4 vs. 20.8 ± 8.9, *t* = −2.2, *df* = 102, *p* = 0.034) and worse (higher) CGI-BP-OS (2.6 ± 0.6 vs. 1.9 ± 0.8, *t* = 4.7, *df* = 103, *p* < 0.001) (Table 1). Although current irritability was inversely associated with history of psychosis (31.6 vs. 55.2 %, *χ*^2^ = 5.5, *df* = 1, *p* = 0.025) and prior psychiatric hospitalization (31.6 vs. 55.2 %, *χ*^2^ = 5.5, *df* = 1, *p* = 0.025), both of these relationships were mediated by the association of bipolar II subtype with current irritability. In contrast, no assessed demographic parameter and no other illness characteristic in Table 1 were significantly associated with current irritability among recovered patients.

### Demographics and illness characteristics/current mood symptoms in depressed patients with vs. without current irritability

Among currently depressed patients, presence compared to absence of current irritability was significantly more common (68.6 vs. 31.4 % had and lacked current irritability, respectively, binomial test *p* < 0.0001). Current irritability was significantly associated with higher rates of lifetime anxiety disorder (84.8 vs. 60.4 %, *χ*^2^ = 11.1, *df* = 1, *p* = 0.002), bipolar II disorder (69.5 vs. 43.8 %, *χ*^2^ = 9.2, *df* = 1, *p* = 0.004), having ≥one first degree relative with a mood disorder (65.7 vs. 45.8 %, *χ*^2^ = 5.4, *df* = 1, *p* = 0.022), episode accumulation (≥10 prior mood episodes, 78.1 vs. 58.7 %, *χ*^2^ = 5.8, *df* = 1, *p* = 0.027), and current euphoria (34.3 vs. 14.6 %, *χ*^2^ = 6.3, *df* = 1, *p* = 0.012), irritability (by definition 100.0 vs. 0.0 %, *χ*^2^ = 153.0, *df* = 1, *p* < 0.0001), and anxiety (86.7 vs. 68.8 %, *χ*^2^ = 6.9, df = 1, *p* = 0.014), as well as earlier onset age (15.6 ± 6.3 vs. 20.5 ± 9.5, *t* = −3.8, *df* = 151, *p* < 0.001) (Table 1). The inverse relationship between current irritability and prior psychiatric hospitalization (21.0 vs. 37.5 %, *χ*^2^ = 4.7, *df* = 1, *p* = 0.047) was mediated by the association of bipolar II subtype with current irritability. In addition, although current irritability was significantly associated with a lower rate of having a college degree (38.1 vs. 66.7 %, *χ*^2^ = 10.8, *df* = 1, *p* = 0.002), all significant findings in Table 1 remained so after adjusting for education. In contrast, no other assessed demographic parameter and no other illness characteristic in Table 1 were significantly associated with current irritability among depressed patients.

### Current irritability in relationship to time to and frequency of depressive recurrence

**Fig. 1:**
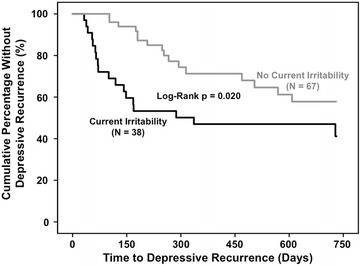
Current irritability associated with hastened depressive recurrence in bipolar disorder. Two-year survival analysis of time to depressive recurrence in recovered bipolar disorder patients indicated significantly hastened depressive recurrence in patients with (*N* = 38, *black line on bottom*) vs. without (*N* = 67, *gray line on top*) current irritability (Log-Rank *p* = 0.020). Current irritability was also significantly associated with hastened depressive recurrence using Cox Proportional Hazard analysis (HR = 2.1; 95 % CI 1.1–4.2; *p* = 0.024). History of anxiety disorder (HR = 3.8, *p* = 0.002) and prior year rapid cycling (HR = 2.6, *p* = 0.052) drove and history of psychosis (HR = 0.38, *p* = 0.023) attenuated hastened depressive recurrence in patients with vs. without current irritability

Current irritability was significantly associated with hastened depressive recurrence (Log-Rank *p* = 0.020) in 38 vs. 67 recovered patients with vs. without current irritability (Fig. 1). Current irritability was also significantly associated with hastened depressive recurrence using Cox Proportional Hazard analysis (HR = 2.1; 95 % CI 1.1–4.2; *p* = 0.024). Hastened depressive recurrence among patients with current irritability was driven by lifetime anxiety disorder (HR = 3.8; 95 % CI 1.6–9.1; *p* = 0.002) and prior year rapid cycling (HR = 2.6; 95 % CI 0.99–6.9; *p* = 0.052), and attenuated by history of psychosis (HR = 0.38; 95 % CI 0.17–0.88; *p* = 0.023). The Kaplan–Meier estimated depressive recurrence rate was only non-significantly higher among patients with (58.9 %; 95 % CI 40.3–77.5 %) compared to without (42.2 %; 95 % CI 25.7–58.7 %) current irritability. Although recovered patients with compared to without current irritability overall had hastened depressive recurrence, as suggested by visual inspection of Fig. 1, patients with current irritability had depressive recurrence significantly less often after compared to before day 180. In other words, current irritability’s association with hastened depressive recurrence was significant up to 180 days (HR = 5.3; 95 % CI 2.0–3.6; *p* = 0.001), but became non-significant after 180 days (HR = 0.50; 95 % CI 0.14–1.8; *p* = 0.29). This appeared to contribute to the black (with current irritability) curve flattening and converging with the still declining gray (without current irritability) curve after 180 days (Fig. 1).

In contrast, associations between current irritability and times to mood elevation recurrence (Log-Rank *p* = 0.18, HR = 0.48; 95 % CI 0.16–1.4; *p* = 0.19, in 38 vs. 67 recovered patients with vs. without current irritability, not illustrated) and any mood episode recurrence (Log-Rank *p* = 0.30, HR = 1.3; 95 % CI 0.78–2.3; *p* = 0.30, in 38 vs. 67 recovered patients with vs. without current irritability, not illustrated) were non-significant.

Upon examining current irritability thresholded for having occurred on at least four of the prior 10 days and at least seven of the prior 10 days, the relationships between current irritability and times to depressive recurrence were non-significant.

### Current irritability in relationship to time to and frequency of depressive recovery

**Fig. 2:**
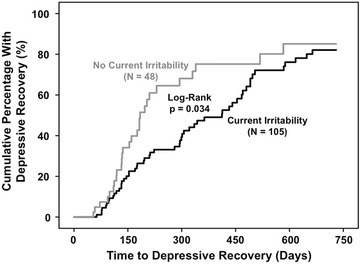
Current irritability associated with delayed depressive recovery in bipolar disorder. Two-year survival analysis of time to depressive recovery in depressed bipolar disorder patients indicated significantly delayed depressive recovery in patients with (*N* = 105, *black line on right*) vs. without (*N* = 48, *gray line on left*) current irritability (Log-Rank *p* = 0.034). Current irritability was also significantly associated with delayed depressive recovery using Cox Proportional Hazard analysis (HR = 0.62; 95 % CI 0.39–0.97; *p* = 0.036). No other assessed parameter mediated delayed depressive recovery in patients with vs. without current irritability

Current irritability was significantly associated with delayed depressive recovery (Log-Rank *p* = 0.034) in 105 vs. 48 depressed patients with vs. without current irritability (Fig. 2). Current irritability was also significantly associated with delayed depressive recovery using Cox Proportional Hazard analysis (HR = 0.62; 95 % CI 0.39–0.97; *p* = 0.034). No other assessed parameter mediated the relationship between current irritability and delayed depressive recovery. Median time to recovery was more than twice as long among patients with current irritability (412 days; 95 % CI 295.3–528.7) compared to those without current irritability (184 days; 95 % CI 154.7–213.3). The Kaplan–Meier estimated 2-year overall depressive recovery rate was 83.0 %, and was very similar and only non-significantly lower among patients with (82.1 %; 95 % CI 71.9–92.3 %) vs. without (85.1 %; 95 % CI 71.0–99.2 %) current irritability. As expected, the Kaplan–Meier estimated 1-year overall depressive recovery rate was lower, at only 57.2 %. Although depressed patients without compared to with current irritability overall took less than half as long to recover, as suggested by visual inspection of Fig. 2, patients without current irritability recovered significantly less often in the second compared to the first year. In other words, the relationship between current irritability and delayed depressive recovery was significant up to 360 days (HR = 0.47; 95 % CI 0.29–0.78; *p* = 0.004), but became non-significant after 360 days (HR = 2.2; 95 % CI 0.53–9.8; *p* = 0.27). This appeared to contribute to the gray (without current irritability) curve flattening and converging with the still rising black (with current irritability) curve in the second year (Fig. 2).

In addition, current irritability was significantly related to delayed recovery from any mood episode (Log-Rank *p* = 0.029, HR = 0.63; 95 % CI 0.41–0.96; *p* = 0.031, in 147 vs. 59 patients in a current mood episode with vs. without current irritability, not illustrated). However, the association between current irritability and time to mood elevation recovery (Log-Rank *p* = 0.35, HR = 0.57; 95 % CI 0.17–1.9; *p* = 0.36, in 42 vs. 11 manic/hypomanic/mixed patients with vs. without current irritability, not illustrated) was non-significant.

Current irritability dichotomized for having occurred on at least four and seven of the prior 10 days was not and was significantly associated with delayed recovery from depression, respectively.

## Discussion

In this prospective study, we found that current irritability had statistically significant associations with both hastened depressive recurrence and delayed depressive recovery. Whereas the relationship between current irritability and hastened depressive recurrence was driven by history of anxiety disorder and prior year rapid cycling, and attenuated by history of psychosis, that between current irritability and delayed depressive recovery was not significantly mediated by any other assessed parameter. The unfavorable relationship between current irritability and illness severity may have been even more robust among currently recovered vs. currently depressed patients, considered not only at baseline (significantly associated with 68.4 vs. 36.8 % unfavorable illness characteristics/current mood symptoms) but also possibly longitudinally (depressive recurrence hastened with *p* = 0.020 vs. depressive recovery delayed with *p* = 0.034).

Our rates of depressive recurrence and recovery were comparable to those found in other studies. A review of naturalistic studies with 2–2.5 years of follow-up determined a mean depressive recurrence rate of 55.7 % (Vazquez et al. 2015), as compared to 48.2 % within 2 years in the current study. Moreover, a prior study by Otto et al. reported that 58.4 % of patients recovered from bipolar depression within 1 year (Otto et al. 2006), while 57.2 and 83.0 % of our patients achieved recovery within one or 2 years, respectively.

The association between current irritability and hastened depressive recurrence suggests that irritability could have adverse longitudinal effects upon depressive recurrence similar to bipolar disorder residual mood symptoms considered in aggregate. Residual affective symptoms considered in aggregate not only predicted mood episode recurrence (Judd et al. 2008; Keller et al. 1992; Perlis et al. 2006), but also were associated with additional unfavorable illness characteristics, such as suicidal behavior and Axis I comorbidity (MacQueen et al. 2003; Parmentier et al. 2012). Furthermore, although irritability has been more extensively studied in the context of mania, hypomania, and mixed depression (Balázs et al. 2006; Benazzi 2007; Benazzi and Akiskal 2003b; Goldberg et al. 2009; Winokur and Tsuang 1975), the impact of irritability during euthymia is becoming increasingly apparent (Parmentier et al. 2012). Our findings extend prior observations by suggesting that current irritability is associated with hastened depressive recurrence. Indeed, irritability may be similar to residual mood elevation symptoms considered in aggregate, as Perlis and associates found that residual mood elevation symptoms considered in aggregate (which can include irritability) predicted hastened depressive recurrence (Perlis et al. 2006).

Similarly, the relationship between current irritability and delayed depressive recovery indicated that irritability could have important longitudinal implications for acute bipolar depression. Although irritability does not currently count as one of the “non-overlapping” mood elevation symptoms towards depression with mixed features in adults (American Psychiatric Association 2013), previous studies have demonstrated that irritability is highly prevalent in, and is independently related to, mixed depression, which in turn can entail delayed recovery (Balázs et al. 2006; Benazzi and Akiskal 2003a, 2005; Pae et al. 2012; Perugi et al. 2015).

The relationships between current irritability and both depressive recurrence and depressive recovery are consistent with the possibility of a particularly strong connection between current irritability and longitudinal depressive burden. Shah and associates found that current irritability was the only assessed parameter to be significantly associated with not only depressive recurrence but also depressive recovery (Shah et al. 2016 in review). Thus, although current irritability is robustly related to current anxiety (Yuen et al. 2016), the former may prove to be an even stronger predictor of delayed depressive recovery. Clearly, there is a need for additional study of the role of current irritability in longitudinal depressive severity.

For example, further studies are needed to determine the direction of causality in the associations between current irritability and hastened depressive recurrence and delayed depressive recovery. Treatment studies targeting irritability could assess its effect on bipolar course. Although this relationship remains to be examined in more detail, prior studies have indicated that adjunctive quetiapine (Sokolski and Denson 2003) and adjunctive gabapentin (Vieta et al. 2000) can relieve not only depressive symptoms but also irritability. In addition, studies are needed to further characterize the contributions of lifetime history of anxiety disorder, prior year rapid cycling, and lifetime history of psychosis to the relationship between current irritability and depressive recurrence. Lifetime history of anxiety disorder may be of particular interest, as studies have demonstrated it to be robustly associated with both current irritability (Yuen et al. 2016) and depressive recurrence (Shah et al. 2016 in review).

Our study has the strengths of assessing relationships between current irritability and not only hastened depressive recurrence but also delayed depressive recovery, using validated instruments to assess diagnosis and longitudinal course, and having substantial numbers of both well-characterized recovered (*N* = 105) and depressed (*N* = 153) BD patients. In addition, our current irritability hastening depressive recurrence finding was not merely the result of subsequent mood elevation episodes leading to depression, as patients with subsequent manic, hypomanic, and mixed episodes were censored.

Limitations of this study include the use of a sample referred to a suburban Northern California BD specialty clinic, limiting the generalizability of our findings in our relatively affluent, well educated but relatively underemployed, predominantly female sample of BD patients with medical insurance. Moreover, our study was limited by: (1) the absence of use of a validated reliable irritability rating scale (e.g., the Buss-Durkee hostility inventory); (2) the lack of determination of the mechanism(s) of loss of statistical significance (ranging from reduced reliability to reduced statistical power) when increasing the threshold for current irritability from any to 4 or 7 days; (3) the possibility that mild mixed depressive symptoms could have been detected as current irritability and hence contributed to poorer prognosis—indeed in the entire sample, current irritability was associated with subsyndromal depressive symptoms (Yuen et al. 2016); and (4) the absence of use of a temperament assessment, limiting insight into character and pre-morbid functioning. Additionally, our sample size, though substantial, had insufficient statistical power to be able to adequately assess relationships between current irritability and times to mood elevation recurrence/recovery. Furthermore, mood episode episode/recovered status duration prior to enrollment was not included in our analyses of mood episode recurrence and recovery. Another limitation is the open naturalistic treatment design, in which patients received diverse uncontrolled (albeit guideline-informed, measurement- and evidence-based) therapies. Finally, we did not correct for multiple comparisons, which particularly limited interpretation of findings with *p* values between 0.05 and 0.01. However, this liberal statistical approach increased assay sensitivity with respect to our ability to detect relationships between current irritability and depressive recurrence and recovery.

Nevertheless, we contend that our observation of associations between current irritability and hastened depressive recurrence as well as delayed depressive recovery suggests that irritability may be an important indicator of longitudinal depressive burden in BD. Given the worse outcomes associated with bipolar depression and irritability, further examination of these relationships is warranted to better understand their mechanisms and clinical implications.

## Abbreviations

BD: bipolar disorder
CGI-BP-OS: clinical global impression for bipolar disorder-overall severity
CI: confidence interval
CMF: clinical monitoring form
df: degrees of freedom
HR: hazard ratio
MINI: Mini International Neuropsychiatric Interview
SCID for DSM-IV: structured clinical interview for the fourth edition of the diagnostic and statistical manual of mental disorders
SD: standard deviation
SPSS: statistical package for the social sciences
STEP-BD: systematic treatment enhancement program for bipolar disorder
